# An Underestimated Cause of Hypercalcemia: A Case Report of Milk-Alkali Syndrome

**DOI:** 10.7759/cureus.93040

**Published:** 2025-09-23

**Authors:** Md Anik Rahman, Farhan Baig, Palak Sharma, Md Khalilur Rahman, Mohammad F Chowdhury, Vinay Gottam, Gihan Musa

**Affiliations:** 1 Acute Medicine, Calderdale and Huddersfield NHS Foundation Trust, Huddersfield, GBR; 2 General Internal Medicine, Calderdale and Huddersfield NHS Foundation Trust, Huddersfield, GBR; 3 Acute Medicine, Huddersfield Royal Infirmary, Huddersfield, GBR; 4 Internal Medicine, Calderdale and Huddersfield NHS Foundation Trust, Huddersfield, GBR

**Keywords:** acute renal failure, calcium carbonate, case report, hypercalcemia, metabolic alkalosis, milk-alkali syndrome

## Abstract

Milk-alkali syndrome (MAS) was first described as an adverse effect of the Sippy regimen, which contained large amounts of sodium bicarbonate, creating a clinical scenario in which hypercalcemia, metabolic alkalosis, and acute renal failure could develop simultaneous features characteristic of MAS. The syndrome has resurged due to widespread calcium supplement use. A 50-year-old Caucasian female was referred to the emergency department by her general physician after an incidental finding of hypercalcemia during evaluation for fatigue. She was diagnosed with acute renal failure in association with elevated calcium levels. After excluding other causes (e.g., hyperparathyroidism, malignancy, multiple myeloma, and familial hypocalciuric hypercalcemia), detailed history and labs confirmed MAS. Intravenous hydration and discontinuation of calcium carbonate normalized her renal function and calcium levels. The case highlights the importance of regular enquiry into diet and consumption of over-the-counter supplements in the assessment of hypercalcemia.

## Introduction

Milk-alkali syndrome (MAS) is categorized by acute renal failure, metabolic alkalosis, and hypercalcemia, resulting from excessive intake of calcium and absorbable alkali [1-3]. First identified in the early 1900s during the era when milk and alkali were standard peptic ulcer treatments, MAS became less common with the advent of modern ulcer medications like proton pump inhibitors and histamine-2 blockers [4]. However, over the past two decades, MAS has re-emerged, largely due to the widespread use of calcium-containing supplements [5]. Calcium carbonate is one of the most commonly used over-the-counter preparations for the management of hypocalcemia, dyspepsia, and osteoporosis, and in dialysis patients, transplant recipients, and postmenopausal women [4]. Pregnant women are also at heightened risk because hyperemesis gravidarum causes fluid loss and alkalosis, compounded by naturally increased calcium absorption during pregnancy [6]. Over the last several years, the clinical spectrum of MAS has expanded and now exhibits mild, asymptomatic hypercalcemia to fatal renal and metabolic complications [7]. The syndrome is not well identified in clinical practice because it presents with similar symptoms to other causes of hypercalcemia that have higher prevalence, including primary hyperparathyroidism and malignancy [8]. This diagnosis difficulty may postpone proper care to result in deteriorating renal deprivation and high morbidity. In addition, increased availability of self-prescribed calcium and vitamin D supplementation (without supervision) has also led to a consistent rise in disease prevalence [9]. Such a tendency represents a strong public health issue, since patients might know nothing about the risks of excessive consumption, especially when combined with other risk factors, including dehydration, the use of other diuretics, or underlying kidney disease. Clinicians should then have a high index of suspicion of MAS, particularly in people who are at risk of overusing calcium. Although it has a long history, MAS is a clinically neglected factor in the causes of hypercalcemia in the contemporary clinical setting. A significant proportion of patients go through a lengthy process of assessment of more etiological causes than MAS, extending the diagnosis and treatment time. This shows the importance of continuous sensitization of clinicians on supplement-related metabolic imbalances. The purpose of this case report is to shed light on MAS as an often under-recognized etiology of hypercalcemia, the importance of prompt treatment, and to present the clinical course and management approaches by providing an example of our patient.

## Case presentation

Case history

A 50-year-old Caucasian woman was referred to the emergency department by her general practitioner following an incidental finding of hypercalcemia. She reported a two-week history of fatigue, abdominal discomfort, intermittent pain, constipation, vomiting episodes, and myalgias. She denied weight loss, night sweats, or fever. Past medical history included hypoparathyroidism following thyroidectomy nine years prior for autoimmune thyrotoxicosis, mixed anxiety and depressive disorder, osteoarthritis of the knee, hypertension, paroxysmal atrial fibrillation, and a previous episode of drug overdose. She was on levothyroxine, alfacalcidol 1 microgram, calcium carbonate replacement for previous episodes of hypocalcemia, along with other medications for comorbidities. She used to drink six pints of milk daily.

Physical examination and investigations

On examination, the patient was hemodynamically stable (afebrile, normotensive). The neurological exam was grossly intact. There was no evidence of an enlarged lymph node or mass lesion elsewhere in the body. She had a dry mucous membrane. She was found to have an unremarkable cardiorespiratory and gastrointestinal examination. There was no evidence of tenderness over the shoulder girdle, pelvic girdle, or muscle on palpation.

Key biochemical parameters are presented in Table 1. The rest of the investigations, including alkaline phosphatase level, angiotensin-converting enzyme level, myeloma screen, all other electrolytes, infection parameters, and thyroid function tests, were within the reference range. A CT scan of the thorax, abdomen, and pelvis was done to identify any mass lesions, but reported normal findings.

**Table 1:** Key laboratory findings during hospitalization for hypercalcemia.

Parameter	Result	Reference range	Interpretation
Adjusted serum calcium	3.37 mmol/L	2.20 – 2.60 mmol/L	High (hypercalcemia)
Inorganic phosphate	1.77 mmol/L	0.80 – 1.50 mmol/L	Elevated
25-hydroxy vitamin D	48 nmol/L	50 – 150 nmol/L (sufficient)	Low/insufficient
Intact parathyroid hormone (PTH)	<0.50 pmol/L	1.95 – 8.49 pmol/L	Low (suppressed)
Parathyroid hormone-related peptide	1 pmol/L	<1.8 pmol/l	Normal
Urine calcium	3 mmol/L	2.5 – 7.5 mmol/L	Normal
Serum creatinine	436 µmol/L	60 – 110 µmol/L	High
Serum urea	14.4 mmol/L	2.5 – 7.8 mmol/L	High
Estimated glomerular filtration rate	9 mL/min/1.73 m²	>90 mL/min/1.73 m²	Reduced
Serum chloride	94 mmol/L	95 – 108 mmol/L	Slightly low
Venous pH	7.46	7.35 – 7.45	Alkalotic
Bicarbonate (HCO₃⁻)	30 mmol/L	22 – 28 mmol/L	High (metabolic alkalosis)
Partial pressure of carbon dioxide (PCO₂)	42 mmHg	35 – 45 mmHg	Normal
Partial pressure of oxygen (PO₂)	100 mmHg	75 – 100 mmHg (arterial)	Normal

Differential diagnosis

Given the history of excessive daily milk intake (six pints), calcium supplementation, and vitamin D replacement, combined with hypercalcemia, acute kidney injury, metabolic alkalosis, and suppressed parathyroid hormone (PTH), MAS was diagnosed after excluding other causes of hypercalcemia.

Treatments and outcomes

**Figure 1:**
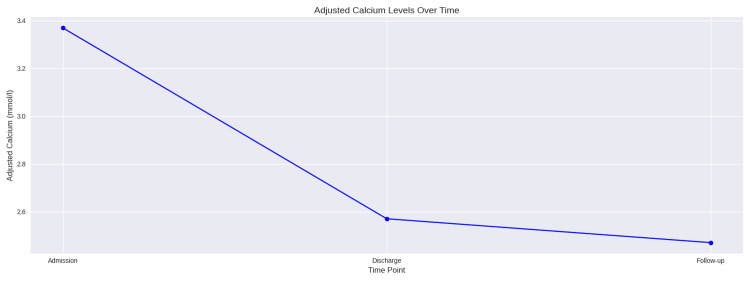
Treatment response to high calcium in milk-alkali syndrome.

She was treated aggressively with 4 L of intravenous crystalloid fluids daily with strict input/output charting. The calcium supplement, alfacalcidol, and ramipril were stopped from the medication chart. After 10 days of hospital stay, she was discharged from the hospital with resolution of hypercalcemia and acute kidney injury. She did not require any form of dialysis for acute kidney injury. Calcium was coming down smoothly after intravenous fluid management and stopping certain medications. During discharge, her calcium level was reduced to 2.57 mmol/L (2.20-2.60 mmol/L). She was discharged with alfacalcidol at a regular dose and a suspended calcium supplement (calcium carbonate) from the medication chart. One month later, she was reviewed in the same-day medical emergency care. Her calcium level was 2.47 mmol/L. Further, she was given regular follow-up in the endocrine clinic for monitoring of nephrocalcinosis via renal ultrasound, given the patient's history of acute kidney injury (Figure 1).

## Discussion

Our patient was taking 3 g of calcium carbonate daily along with alfacalcidol and ramipril, which increased the risk of MAS. Calcium carbonate and alfacalcidol, an active vitamin D analog, supplement intestinal calcium absorption, both of which exacerbate the metabolic derangements of MAS [10], and angiotensin-converting enzyme (ACE) inhibitors, including ramipril, decrease glomerular filtration pressure through efferent arteriolar vasodilation [11]. These factors played a vital role in increasing the risk of MAS, even with low calcium ingestion not exceeding 4 g/day.

MAS was first documented in the early 1900s as a complication of the Sippy regimen (milk and alkali) used for gastric ulcers [4]. It manifests acutely or chronically. Orwoll described its acute phase and severe chronic form [11], detailing its prolonged toxicities: nephrocalcinosis leading to renal failure and band keratopathy (corneal calcium deposits) [12]. This chronic variant is termed "Burnett's Syndrome" [13]. Following the advent of modern peptic ulcer treatments, the syndrome's incidence declined sharply; by 1985, it accounted for <1% of hypercalcemia cases [14]. However, over the past two decades, widespread use of calcium supplements has caused a significant resurgence [5].

MAS has emerged as the third most common cause of hypercalcemia requiring hospitalization, surpassed only by malignancy and primary hyperparathyroidism [5,15]. Its resurgence is largely attributed to the widespread consumption of over-the-counter calcium carbonate supplements, commonly used for conditions such as osteoporosis, dyspepsia, hypocalcemia, and among high-risk groups, including postmenopausal women and patients with chronic kidney disease [4,5]. Although MAS is typically associated with calcium intakes exceeding 4 g/day, cases have been documented with lower intakes of 1.0-1.5 g/day in individuals with predisposing factors, including eating disorders, hyperemesis gravidarum, or renal impairment [4,16]. The diagnosis is based on the characteristic triad of PTH, i.e., independent hypercalcemia, metabolic alkalosis, and renal insufficiency, often accompanied by normal or low serum phosphate levels.

Pathophysiologically, hypercalcemia stimulates calcium-sensing receptors (CaSRs) in the ascending loop of Henle [17]. This increases calcium excretion but decreases sodium chloride reabsorption, causing volume depletion and hypochloremic metabolic alkalosis. Concurrent CaSR activation in the distal convoluted tubules suppresses aquaporin-2 expression, inducing nephrogenic diabetes insipidus and further reducing water reabsorption [18]. The resulting alkalosis increases tubular calcium reabsorption and decreases excretion, worsening hypercalcemia [13,19]. Hypercalcemia also constricts the afferent arteriole, reduces glomerular filtration rate (GFR), and combines with polyuria and impaired bicarbonate excretion to cause intravascular depletion, ultimately leading to renal failure [4,20].

Symptoms of MAS are related to the severity of hypercalcemia and renal failure. Often, it remains unrecognized, and the clinician requires a high suspicion score to diagnose MAS. Physicians should be aware of incidental hypercalcemia to diagnose MAS if other differentials are excluded. Key differential diagnoses for hypercalcemia include familial hypocalciuric hypercalcemia (FHH), malignancy, primary hyperparathyroidism, medication-induced causes (e.g., thiazides and lithium), granulomatous diseases (e.g., sarcoidosis), hyperthyroidism, adrenal insufficiency, acromegaly, and vitamin D toxicity. Confirming MAS requires a thorough history, physical exam, and targeted labs.

Management of hypercalcemia in MAS is supportive, including intravenous hydration and cessation of calcium/alkali intake. Acute MAS typically resolves rapidly following discontinuation of calcium/alkali sources. Conversely, chronic MAS recovery is slower and often complicated by metastatic calcification, including band keratopathy and nephrocalcinosis [21]. Loop diuretics may be used occasionally but are unsupported by evidence and risk worsening renal function; thus, their use is discouraged. Supportive care remains the cornerstone of therapy [22]. A distinctive feature is rebound hypocalcemia following treatment with bisphosphonates, and they are also not recommended [23].

After the treatment and one-month follow-up, our patient experienced a positive clinical outcome, which included normalization of calcium and renal functioning after the early identification of MAS and the initiation of calcium/alkali source withdrawal, along with aggressive fluid management. Calcium carbonate, alfacalcidol, and ramipril were avoided during hospitalization as they have contributory effects on the escalation of hypercalcemia and renal dysfunction. After discharge, the patient's serum calcium levels were maintained, with no signs of recurrence of symptoms, which highlights the efficiency of conservative management and the significance of patient education to avoid re-exposure. It is important to be aware of MAS to avoid the use of bisphosphonate, which is a common practice in the treatment of hypercalcemia, to avoid complications like hypocalcemia and worsening renal function, as the prevalence of MAS is 9-12% among inpatients [24].

## Conclusions

MAS should not be underestimated by physicians, as it is now recognized as the third most common cause of hypercalcemia-related hospital admissions. A high index of suspicion and awareness is needed to diagnose MAS due to the potential adverse outcome. Bisphosphonate has no proven benefit in treating hypercalcemia in MAS and can cause rebound hypocalcemia. Calcium-lowering medication can induce hypocalcemia, necessitating calcium replacement, in contrast to other causes of hypercalcemia. Prompt clinical judgment is important because early identification and removal of the offending agent are often enough to reverse the condition. Education on the dangers of unsupervised calcium supplementation to the patient is also paramount in terms of preventing relapse. The case highlights the importance of regular enquiry into diet and consumption of over-the-counter supplements in the assessment of hypercalcemia. Routine screening for supplement use in hypercalcemia workups could reduce diagnostic delays and healthcare costs, particularly in primary care settings.
